# Psychological Impact of Aggressive Incidents on Healthcare Professionals in Psychiatric Wards: An Observational Study

**DOI:** 10.1192/j.eurpsy.2026.11511

**Published:** 2026-07-31

**Authors:** V. Caivano, V. Pagano, A. Mazza, A. Savastano

**Affiliations:** 1 Department of Mental Health - SPDC Salerno, ASL Salerno, Salerno; 2 Department Of Psychiatry, University of Campania “Luigi Vanvitelli”, Naples, Italy

## Abstract

**Introduction:**

In recent years, the number of aggressive incidents in psychiatric wards has progressively increased, representing one of the main challenges to staff safety and the overall quality of the work environment. Ensuring protection and well-being for healthcare professionals is not merely an organizational requirement, but a key factor in the effectiveness of care and in safeguarding the mental health of those working in such high-demand settings.

**Objectives:**

This study aimed to assess the prevalence of aggression among healthcare professionals in the Campania region and its psychological impact on them.

**Methods:**

A cross-sectional observational study was conducted using an ad hoc questionnaire administered to psychiatric ward staff in the Campania region. The questionnaire collected sociodemographic and professional data, experiences of aggression, and job satisfaction. Psychological distress was assessed using the Depression Anxiety Stress Scales–21 items (DASS-21).

**Results:**

Fifty healthcare professionals were enrolled in the study: 42.0% were medical or psychological staff, and the remainder were nursing or other healthcare workers. Females comprised 54.0% of the sample, with a mean age of 44.4±10.0 years. Overall, 94.0% reported experiencing at least one aggressive episode, either verbal (82.0%) and/or physical (52.0%), while 78.0% reported feeling unsafe at work. Pearson correlation analyses revealed a significant inverse relationship between age and DASS-21 anxiety (r=-0.294, p<0.05) and stress (r=-0.289, p<0.05) subscales, as well as between years of psychiatric ward experience and the anxiety subscale (r=-0.315, p<0.05). Women had significantly higher mean scores on the DASS-21 anxiety and depression subscales compared to men (p<0.01). Participants considering leaving their job exhibited significantly higher mean scores across all DASS-21 subscales (p<0.05).

**Conclusions:**

The findings confirm the high prevalence of aggression in psychiatric wards and its psychological impact on staff, with greater levels of distress among women and less experienced professionals, to the point of fostering intentions to leave their job. These results underscore the urgent need for targeted interventions in prevention, support, and training, as well as a critical reassessment of the effectiveness of current safety measures.

**Disclosure of Interest:**

None Declared

